# Complete Genome Sequences of Chop, DelRio, and GrandSlam, Three *Gordonia* Phages Isolated from Soil in Central Arkansas

**DOI:** 10.1128/mra.00023-23

**Published:** 2023-04-10

**Authors:** Heidi N. Mathes, Elijah I. Christenson, John H. Crum, Emme M. Edmondson, Kassidy E. Gray, Luke W. Lawson, Lauren E. Lee, Michael P. Lee, Jackson A. Lipscomb, Morgan E. Masengale, Hannah G. Matthews, Charles M. McClain, Tuesday N. Melton, Trace H. Morrow, Alexis M. Perry, David R. Rainwater, Grace E. Renois, Maryann F. Rettig, Duncan C. Troup, Allie J. Wilson, Nathan S. Reyna, Ruth Plymale

**Affiliations:** a Department of Biology, Ouachita Baptist University, Arkadelphia, Arkansas, USA; b UAMS College of Medicine, University of Arkansas for Medical Sciences, Little Rock, Arkansas, USA; c Nursing, Encompass Health Rehabilitation Hospital, Texarkana, Texas, USA; d Speech Therapy, InnovAge Total Long Term Care, Loveland, Colorado, USA; e Louisiana State University School of Dentistry, New Orleans, Louisiana, USA; DOE Joint Genome Institute

## Abstract

Chop, DelRio, and GrandSlam are phage with a *Siphoviridae* morphotype isolated from soil in Arkansas using the host Gordonia terrae 3612. All three are temperate, and their genomes share at least 96% nucleotide identity. These phage are assigned to cluster DI based on gene content similarity to other sequenced actinobacteriophage.

## ANNOUNCEMENT

We report on three bacteriophage, Chop, DelRio, and GrandSlam, that were isolated on Gordonia terrae 3612 (1). These phage also infect Gordonia rubripertincta NRRL B-16540 at a much reduced efficiency of plating, suggesting a potentially expanding host range (2).

**FIG 1:**
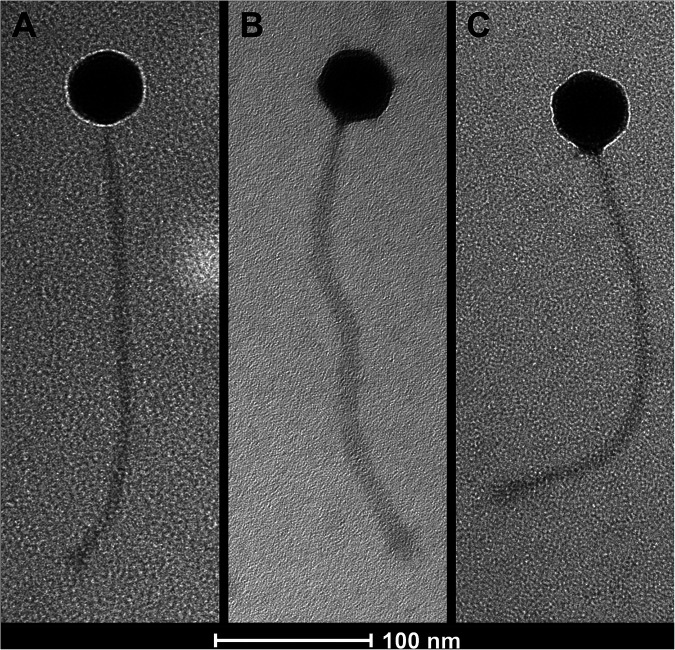
Transmission electron micrographs of *Gordonia* phages Chop (A), DelRio (B), and GrandSlam (C). Phage lysates were negatively stained with 1% uranyl acetate and viewed using a Tecnai F20 transmission electron microscope, with images taken at 80 kV and 80,000× on a Gatan Eagle camera.

All three phage were isolated from soil (3), with Chop isolated from garden soil, DelRio from the bank of the Caddo River, and GrandSlam from a pitching mound (see Table 1 for global positioning system [GPS] coordinates). Briefly, soil samples were washed in peptone-yeast extract-calcium (PYCa) medium, and the wash was filtered through a 0.22-μm filter, then combined with Gordonia terrae 3612, and incubated with shaking for 3 or 4 days at 30°C. The culture was then spun, the supernatant was plated in PYCa top agar with *G. terrae* 3612, and the plates were incubated at 30°C for 3 or 4 days. Four rounds of plaque purification were performed for Chop, and three rounds were performed for DelRio and GrandSlam. After incubation for 3 to 4 days at 30°C, phage replication produced turbid plaques with a diameter of 1.5 to 2 mm for Chop, 4 mm for DelRio, and 3 mm for GrandSlam. Viewed by negative-stain transmission electron microscopy, all three phage showed a *Siphoviridae* morphotype (Fig. 1). Capsid diameters and tail lengths were measured with ImageJ v1.53k (4) and are listed in Table 1.

**TABLE 1:** Isolation details, sequencing results, and genome and virion characteristics of Chop, DelRio, and GrandSlam

Phage name	Date collected	Location (GPS coordinates)	Avg coverage (×)	No. of reads (thousands)	Genome size (bp)	Genome end	GC content (%)	No. of genes	Capsid diam (nm ± SD) (no. of particles)	Tail length (nm ± SD) (no. of particles)
Chop	September 2021	34.3099N, 93.1514W	2,221	757.0	50,919	3′ 10-base extension	67	76	46.3 ± 1.7 (3)	277.4 ± 6.9 (3)
DelRio	August 2017	34.1768N, 93.0714W	594	940.4	50,961	3′ 10-base extension	67	75	61.1 ± 11.4 (3)	275.5 ± 12.2 (3)
GrandSlam	August 2017	34.2958N, 92.4219W	2,034	915.3	50,919	3′ 10-base extension	67	76	46.8 (1)	279.6 (1)

Phage lysates were concentrated by pelleting and resuspending phage following polyethylene glycol precipitation (3). DNA was extracted using the Promega Wizard DNA cleanup kit, prepared for sequencing using the New England Biolabs (NEB) Ultra II Library kit, and sequenced on Illumina MiSeq (v3 reagents); 150-bp single-end reads yielded 2,221-fold (Chop), 594-fold (DelRio), and 2,034-fold (GrandSlam) genome coverage (Table 1). Raw reads were assembled using Newbler v2.9 (5), assembly completeness was determined using Consed v29 (http://www.phrap.org/consed/consed.html), and ends were identified using PAUSE (https://cpt.tamu.edu/computer-resources/pause/), all with default settings. Despite their geographic isolation, the three genomes are remarkably similar, sharing at least 96% nucleotide identity by BLASTn (6). Based on gene content similarity to other actinobacteriophage (https://phagesDB.org/), all three phage were assigned to cluster DI (7, 8). The genomes all have 67% GC content and 3′ single-stranded genome ends (5′-TGCCGCGGTA-3′).

The genomes were autoannotated with DNAMaster v2700 (http://cobamide2.bio.pitt.edu) using GLIMMER v3.02 (9) and GeneMark v2.5 (10) and manually refined using Phamerator v467 (11), Aragorn v1.2.41 (12), and PECAAN v20211202 (https://discover.kbrinsgd.org). Using BLASTp (6) and HHpred (13), putative functions were assigned to 40 of 76 annotated genes in Chop, 37 of 75 genes in DelRio, and 37 of 76 genes in GrandSlam. The majority of genes are transcribed rightward with a small number of leftward-transcribed genes in the center of each genome. The leftward-transcribed genes follow the annotated lysis cassette and include a predicted HicAB toxin-antitoxin system and putative integrase and immunity repressor. These, with observed plaque turbidity and isolation of a verified DelRio lysogen, support Chop, DelRio, and GrandSlam as temperate phage.

### Data availability.

Chop GenBank and SRA accession numbers are ON637763 and SRX14443491, respectively. DelRio GenBank and SRA accession numbers are MH509446 and SRX5282532, respectively. GrandSlam GenBank and SRA accession numbers are MK967392 and SRX14443509, respectively.
